# Incidence and Risk Factors of Hyperuricemia among 2.5 Million Chinese Adults during the Years 2017–2018

**DOI:** 10.3390/ijerph18052360

**Published:** 2021-02-28

**Authors:** Ruiqi Shan, Yi Ning, Yuan Ma, Xiang Gao, Zechen Zhou, Cheng Jin, Jing Wu, Jun Lv, Liming Li

**Affiliations:** 1 Department of Epidemiology and Biostatistics, School of Public Health, Peking University Health Science Center, Beijing 100191, China; ruiqi0119@bjmu.edu.cn (R.S.); 2011110141@bjmu.edu.cn (Z.Z.); 15527305167@163.com (J.W.); lvjun@bjmu.edu.cn (J.L.); 2 Meinian Public Health Institute, Peking University Health Science Center, Beijing 100191, China; 3 Meinian Institute of Health, Beijing 100191, China; yuan.ma@meinianresearch.com (Y.M.); cheng.jin@meinianresearch.com (C.J.); 4 Department of Nutritional Sciences, The Pennsylvania State University, University Park, PA 16802, USA; xxg14@psu.edu

**Keywords:** hyperuricemia, epidemiology, incidence

## Abstract

*Objective***:** To assess the incidence and risk factors of hyperuricemia among Chinese adults in 2017–2018. *Methods***:** A total of 2,015,847 adults (mean age 41.2 ± 12.7, 53.1% men) with serum uric acid concentrations assayed on at least two separate days in routine health examinations during 2017–2018 were analyzed. Hyperuricemia was defined as fasting serum urate concentration >420 μmol/L in men and >360 μmol/L in women. The overall and sex-specific incidence rate were stratified according to age, urban population size, geographical region, annual average temperature and certain diseases. Logistic regression analyses were performed to explore risk factors associated with hyperuricemia. *Results***:** 225,240 adults were newly diagnosed with hyperuricemia. The age- and sex-standardized incidence rate per 100 person-years was 11.1 (95%CI: 11.0–11.1) (15.2 for men and 6.80 for women). The risk of hyperuricemia was positively associated with younger age, being male, larger urban population size, higher annual temperature, higher body mass index, lower estimate glomerular filtration rate, hypertension, dyslipidemia and fat liver. *Conclusions***:** The incidence of hyperuricemia was substantial and exhibited a rising trend among younger adults, especially among men. Socioeconomic and geographic variation in incidence were observed. The risk of hyperuricemia was associated with estimate glomerular filtration rate, fat liver and metabolic factors.

## 1. Introduction

The prevalence of hyperuricemia has been increasing worldwide, including China [1,2,3]. In the United States, it increased from 18.2% in 1988–1994 [1] to 21.4% in 2007–2008 [2]. It is noteworthy that, in China, it increased from 1.4% to 9.9% for men and 1.3% to 7.0% for women from the 1980s [4] to 2009–2010 [5]. Furthermore, hyperuricemia has already become one of the most common metabolic diseases in China [6]. Accumulating evidence suggests that people with hyperuricemia may have a higher future risk of gout [7], chronic kidney disease [8], hypertension [9], cardiovascular disease [10,11], and mortality [12], via mechanisms such as monosodium urate crystal deposition [13,14], endothelial dysfunction [15,16], intracellular and mitochondrial oxidative stress [17] and stimulation of the intracellular renin angiotensin system, etc. [18], which could pose a serious problem for public health.

However, given the rapidly increasing prevalence and accompanied serious health threats, the epidemiology of hyperuricemia has not been given special attention it deserves [6]. Nationwide Chinese data on the epidemiological of hyperuricemia is still limited, especially about the incidence. 

Recently, an increasing number of the Chinese population have visited health examination centers, which provides an unique opportunity to understand the status of hyperuricemia in China. We thus estimated the incidence of hyperuricemia based on the health examination data of 2,015,847 Chinese adults from 30 provinces, who attended health examinations during 2017–2018. 

## 2. Methods

### 2.1. Study Design and Study Population

This study was conducted using data from adults who participated in the routine health examination in Meinian health-screening centers which cover almost all provinces in mainland China (except Tibet). Those health screening centers equipped with professional and experienced medical teams provided comprehensive health examinations to participants. Moreover, unified standard examination protocol was established in each health examination center to ensure the stability of results.

From 1 January 2017 to 31 December 2018, 2,538,685 adults aged ≥ 18 years with at least two times serum uric acid examinations were included. Participants with missing value or outliers in age, sex and serum uric acid concentrations were excluded. Participants with hyperuricemia at first examination were excluded, leaving a total of 2,015,847 participants for the analysis.

This study has been approved by the Institutional Review Board of Peking University Health Science Center (ID of the approval: IRB00001052-19077). Individual informed consent was waived, as only anonymized data were used in this study.

### 2.2. Assessment of Uric Acid and Hyperuricemia

The procedures of health examination with authority-approved methods and instruments were used in all centers. All laboratories meet the standards of requirements of external quality assessment for clinical laboratories [19]. Blood samples were drawn by venipuncture after 8–12 h of overnight fasting to measure serum uric acid. Serum uric acid was measured using automatic biochemical analyzer using uric acid commercial kit according to the manufacturer’s instruction. Hyperuricemia has been defined as a fasting serum urate concentration >420 μmol/L in men and >360 μmol/L in women. 

### 2.3. Assessment of Other Factors

Demographic data and clinical histories were obtained by trained health professionals through face to face interviews. Participants were categorized into six geographic regions: Northern, Eastern, South-Central, Northeast, Northwest, Southwest [20]. Urban population size were categorized into: more than five million, one to five million, less than one million [21]. The annual average temperature for each province was obtained from the National Meteorological Information Center and was categorized into tertile (tertile1: 3–13 °C; tertile 2: 14–16 °C; tertile 3: 17–25 °C) (http://data.cma.cn, accessed on 28 February 2021).

Anthropometric data such as height, weight, and blood pressure were measured according to standard methods. Hypertension was defined as systolic blood pressure ≥140 mmHg and/or diastolic blood pressure ≥90 mmHg and/or self-reported history of hypertension and/or use of antihypertensive treatment [22]. Body mass index (BMI) was calculated as weight (kg)/height (m)^2^. BMI were categorized into <18.5 kg/m^2^, 18.5 kg/m^2^ ≤ BMI < 24 kg/m^2^, 24 kg/m^2^ ≤ BMI < 28 kg/m^2^, and ≥28 kg/m^2^ respectively [23].

Blood biochemical indexes such as triglycerides (TG), total cholesterol (TC), high-density lipoprotein cholesterol (HDL-C), low-density lipoprotein cholesterol (LDL-C) and serum creatinine were measured using automatic biochemistry analyzer in each center. Dyslipidemia was defined as serum TC concentration of ≥6.2 mmol/L and/or TG ≥ 2.3 mmol/L and/or LDL-C ≥ 4.1 mmol/L and/or HDL-C < 1.0 mmol/L and/or self-reported dyslipidemia [24]. Estimate glomerular filtration rate (eGFR) was predicted by the Chronic Kidney Disease Epidemiology Collaboration equation and were categorized into <60, 60–89, ≥90 mL/min per 1.73 m^2^ [25]. Fat liver disease was detected by ultrasonography performed by experienced technicians. All laboratories meet the standards of requirements of external quality assessment for clinical laboratories [19].

### 2.4. Data Analysis

Incidence of hyperuricemia per 100 person-years was using the number of participants newly diagnosed with hyperuricemia as the numerator and accumulated person-years of total population as the denominator. 

Characteristics of the participants are presented as n (%) for categorical variables. Age- and sex- standardized incidence rates with 95% CI weighted by the standard population of 2010 China Population Sampling Census were calculated. The overall and sex-specific incidence rate were stratified according to age, urban population size, geographical region, annual average temperature, BMI, eGFR, hypertension, dyslipidemia and fat liver. Pearson’s χ^2^ analysis was applied to the comparison of rates. Unadjusted and multivariable logistic regression analyses (adjusting for age, sex, urban population size, geographical region, annual average temperature, BMI, eGFR, hypertension, dyslipidemia and fat liver) were conducted to investigate risk factors for hyperuricemia. Analyses were conducted using SAS, version 9.3 (SAS Institute, Inc., Cary, NC, USA). Figure was drawn using R version 4.0 (http://www.r-project.org/, accessed on 28 February 2021). *p* < 0.05 was considered statistically significant.

## 3. Results

Characteristics of the participants were presented in Table 1. The average age was 41.9 ± 13.0 years for men and was 40.4 ± 12.2 years for women. The proportion of men was 53.1%. 

Age- and sex-standardized incidence of hyperuricemia per 100 person-years was 11.1 (95%CI: 11.0–11.1) for total population. Age-standardized incidence rates per 100 person-years were 15.2 (15.2–15.3) for men, 6.80 (6.74–6.87) for women. The prevalence was higher in cities with larger urban population size (more than five million: 11.5 vs. less than one million: 10.7), and cities with higher annual average temperature (17–25 °C: 11.9 vs. 3–13℃: 10.6). In addition, it was higher in Southwest (14.2) and lower in Northwest (8.64). Besides, it was higher among participants with BMI ≥ 28.0 kg/m^2^ (18.6), eGFR < 60 mL/min per 1.73 m^2^ (17.9), hypertension (13.7), dyslipidemia (12.3) and fat liver disease (16.6) relative to their counterparts. Similar trends were applicable to both men and women (Table 2).

When it comes to provinces, hyperuricemia was highest in Qinghai among men (25.7) and was highest in Yunnan among women (11.9) (Table S2).

In multivariable-adjusted models, younger age (≥60 vs. 18–39 years: OR 0.63,95% CI 0.61–0.64), being male (OR 2.20, 95% CI 2.18–2.23), larger urban population size (more than five vs less than one million: OR 1.11, 95% CI 1.09–1.12), higher annual temperature (17–25℃ vs 3–13℃: OR 1.17, 95% CI 1.14–1.20), higher body mass index (≥28 vs. <18.5 kg/m^2^: OR 2.95, 95% CI 2.85–3.06), lower estimate glomerular filtration rate (≥90 vs. <60 mL/min per 1.73 m^2^: OR 0.45, 95% CI 0.43–0.48), hypertension (OR 1.15,95% CI 1.13–1.16), dyslipidemia (OR 1.19, 95% CI 1.17–1.20) and fat liver (OR 1.55, 95% CI 1.53–1.57) were associated with higher risk of hyperuricemia. Similar associations were found both among men and women. (Table 3)

**Figure 1:**
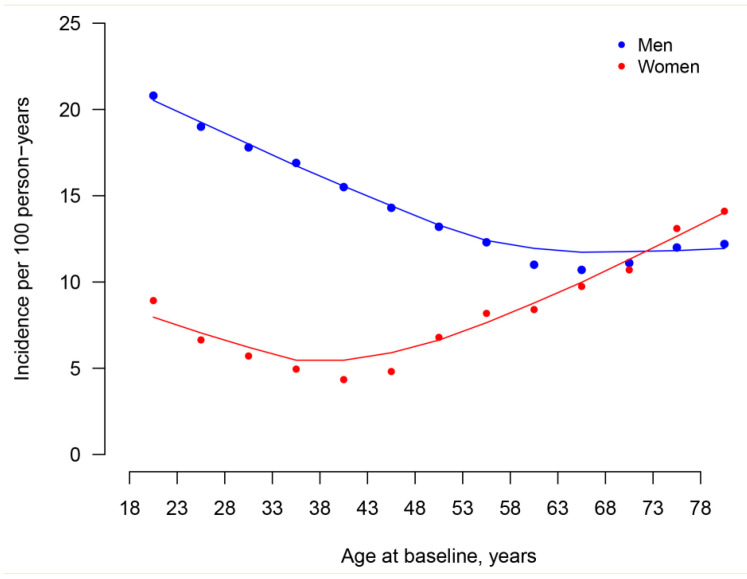
Sex-Specific incidence of hyperuricemia by different age during 2017–2018. *Y*-axis indicates incidence per 100 person-years.

Incidence rates per 100 person-years for men were highest in age 18–22 years (20.8) then gradually decreased and reached the bottom in age 63–67 years (10.7) then gradually increased. Incidence rates per 100 person-years for women were 8.92 in age 18–22 years then gradually decreased and reached the bottom in age 38–42 years (4.34) then gradually increased and reached the peak in age ≥78 years (14.1) (Figure 1, Table S1).

## 4. Discussion

In our study, the incidence rates per 100 person-years of hyperuricemia was 15.2 among men and 6.80 among women. Higher incidence was observed among younger adult, men and in regions with larger urban population size or higher annual average temperature. Higher BMI, lower eGFR, hypertension, dyslipidemia and fat liver were associated with the risk of hyperuricemia.

Plenty of previous studies have focused on the prevalence of hyperuricemia [2,3,5,26,27,28]. The prevalence was reported to be 21.4% in United States during 2007–2008 [2] and 11.9% in Italy during 2009 [3]. In China, the prevalence of hyperuricemia was reported to be 19.4% for men and 7.9% for women during 2000–2014 [26] and 21.6% for men and 8.6% for women during 1995–2009 [27] in two meta-analysis studies. Additionally, Song et al. reported the prevalence was 6.4% in 2010 in a nationally representative sample of Chinese middle-aged and older adults [28]. Besides, Liu et al. reported the prevalence was 8.4% in 2009–2010 in a nationally representative sample of Chinese adult ≥ 18 years old [5]. However, previous researches on the incidence of hyperuricemia remains sparse. The incidence rate was reported to be 4.9 per 100 person-years among South Korean men during 2002–2009 [29] and 3.6–4.9% in Fukushima Prefecture during 2008–2012 [30]. To the best of our knowledge, there are no studies reporting on the nationwide incidence rate in China and it was reported to be 11.1 per 100 person-years in our study. Data from previous studies was scattered across different provinces of China. It was reported that the incidence rate was 4.96 per 100 person-years during 2007–2015 in Shandong [31] and it was 6.9 per 100 person-years in Zhejiang during 2011–2016 [32]. In our study, the incidence rates per 100 person-years were 10.3 in Shandong and 8.5 in Zhejiang, respectively, during 2017–2018, which was higher than previous reports. 

In our study, the incidence was higher among men relative to women, which was in line with previous studies [26,27]. Sex hormones and different lifestyle between men and women may explain the observed sex-difference [33,34]. Several studies illustrated that the prevalence of hyperuricemia increased with age [3,6,27]. However, an inverse trend was observed among men in Henan Rural Cohort Study [35] and a “U” shape was found among men in Korea [36]. In our study, we found that the incidence of hyperuricemia has a trend toward onset at younger age, especially among men. The incidence was highest in youngest age group (age 18–22 years) among men. That is probably due to changing lifestyles accompanied by social economic development such as dining out frequently, adopting unhealthy diets, and increasing social activities involving heavy consumption of alcohol. The incidence also exhibited a trend towards onset at younger age among women, while not as distinctly as for men, and rose greatly after menopause, which may be interpreted as a protective effect of estrogen [33]. A similar trend was found in the study by Cao et al. [31]. 

Further, consistent with previous studies, lower eGFR, obesity, hypertension, dyslipidemia, and fatty liver disease were associated with the risk of hyperuricemia in present study [5,34,37,38,39,40]. As serum uric acid is predominantly cleared by the kidneys, decreased eGFR may be associated with increased serum uric acid levels [41]. Additionally, hyperuricemia is related with endothelial dysfunction [15,16], intracellular and mitochondrial oxidative stress [17], vascular smooth muscle cell proliferation [17], hepatocyte lipid accumulation [42], and stimulation of the intracellular renin angiotensin system, etc. [18], thus hyperuricemia may closely associated with diseases such as obesity, hypertension, dyslipidemia and fatty liver disease which share many similar pathogenic mechanisms [15,16,17,18,40,42,43].

The prevalence of hyperuricemia was higher in cities with larger urban populations. As suggested in previous studies, rapid economic development may be associated with unhealthy lifestyles in developing countries [44]. In addition, the prevalence was higher among provinces with higher annual average temperatures. This is probably a result of accelerated metabolism under higher temperatures [45].

The provinces with the highest hyperuricemia incidence were Qinghai for men and Yunnan for women. Qinghai and Yunnan are both located on a plateau, and residents at high altitude under hypoxic conditions are known to develop adaptive polycythemia [46]. Besides, lactate generated with hypoxia will compete with the excretion of urate in the proximal tubules. In addition, the hypoxia-related increase in hematocrit leads to a decrease in renal plasma flow [47] eventually lead to increasing in uric acid concentration.

Several limitations of this study need to be addressed. First, this was a study based on a health examination population, which may not be generalizable to all Chinese, although the prevalence rates were weighted by the standard population of China to improve the representativeness. Second, information on medical history of hyperuricemia and lifestyle were not available in this study. Nevertheless, the strengths of this study are also worthy of mention. To the best of our knowledge, this is first study to investigate the nationwide incidence rate of hyperuricemia in China. Furthermore, this investigation was based on 2,015,847 Chinese adults covering almost all geographic areas in China, which differ in socioeconomic and geographic features, providing sufficient power to examine the incidence of hyperuricemia at multiple levels.

In conclusion, the incidence of hyperuricemia remains substantial and exhibited a rising trend among younger age groups, especially among men. The incidence was higher in cities with larger urban population size or higher annual average temperature. Besides, higher BMI, lower eGFR, hypertension, dyslipidemia and fat liver disease were all associated with the risk of hyperuricemia. Despite the limitations mentioned before, this study provides valuable information for the prevention of hyperuricemia and on hyperuricemia’s epidemiology and etiology.

## Figures and Tables

**Table 1:** Baseline characteristics of participants.

	Total (*n* = 2,015,847)	Men (*n* = 1,069,622)	Women (*n* = 946,225)
Age, years			
18–39	1,030,779 (51.1)	524,626 (49.0)	506,153 (53.5)
40–59	795,812 (39.5)	436,354 (40.8)	359,458 (38.0)
≥60	189,256 (9.39)	108,642 (10.2)	80,614 (8.52)
Urban population size, million			
Less than one	495,237 (24.6)	270,905 (25.3)	224,332 (23.7)
One to five	787,919 (39.1)	423,803 (39.6)	364,116 (38.5)
More than five	732,691 (36.3)	374,914 (35.1)	357,777 (37.8)
Geographical region			
Northern	270,507 (13.4)	140,328 (13.1)	130,179 (13.8)
Eastern	700,642 (34.8)	365,240 (34.1)	335,402 (35.4)
South-Central	494,709 (24.5)	269,001 (25.1)	225,708 (23.9)
Northeast	181,359 (9.00)	95,210 (8.90)	86,149 (9.10)
Northwest	113,165 (5.61)	65,700 (6.14)	47,465 (5.02)
Southwest	255,465 (12.7)	134,143 (12.5)	121,322 (12.8)
Annual average temperature, °C			
3–13	647,872 (32.1)	342,942 (32.1)	304,930 (32.2)
14–16	733,990 (36.4)	366,669 (34.3)	335,424 (35.4)
17–25	633,985 (31.5)	360,011 (33.7)	305,871 (32.3)
BMI, kg/m^2^			
<18.5	89,777 (5.05)	29,212 (3.11)	60,565 (7.23)
18.5–23.9	932,671 (52.5)	407,357 (43.4)	525,314 (62.7)
24–27.9	578,427 (32.5)	378,760 (40.3)	199,667 (23.8)
≥28.0	176,635 (9.94)	124,176 (13.2)	52,459 (6.26)
eGFR, mL/min per 1.73 m^2^			
<60	11,497 (0.59)	5523 (0.53)	5974 (0.65)
60–89	299,205 (15.2)	180,710 (17.3)	118,495 (12.9)
≥90	1,652,964 (84.2)	856,411 (82.1)	796,553 (86.5)
Hypertension			
Yes	382,003 (19.8)	261,908 (25.6)	120,095 (13.2)
No	1,547,046 (80.2)	760,167 (74.4)	786,879 (86.8)
Dyslipidemia			
Yes	894,034 (44.7)	531,523 (50.1)	362,511 (38.6)
No	1,106,023 (55.3)	529,860 (49.9)	576,163 (61.4)
Fat liver disease			
Yes	606,877 (31.0)	425,478 (40.9)	181,399 (19.7)
No	1,352,360 (69.0)	615,264 (59.1)	737,096 (80.3)

Characteristics of the participants are presented as *n* (%).

**Table 2:** Incidence of hyperuricemia among Chinese health examination adults during 2017–2018.

	Total			Men			Women		
	No. of Cases	Population Denominator, Person-Years	Incidence per 100 Person-Years	No. of Cases	Population Denominator, Person-Years	Incidence per 100 Person-Years	No. of Cases	Population Denominator, Person-Years	Incidence per 100 Person-Years
Total	225,240	2,043,291	11.1 (11.0–11.1)	166,499	1,086,144	15.2 (15.2–15.3)	58,741	957,147	6.80 (6.74–6.87)
Age, years									
18–39	123,231	1,047,453	11.7 (11.6–11.8)	94,234	534,212	17.6 (17.5–17.7)	28,997	513,241	5.65 (5.59–5.71)
40–59	81,649	805,966	9.79 (9.73–9.86)	60,066	442,814	13.6 (13.5–13.7)	21,583	363,151	5.94 (5.87–6.02)
≥60	20,360	189,872	10.6 (10.5–10.8)	12,199	109,117	11.2 (11.0–11.4)	8161	80,754	10.1 (9.9–10.3)
Urban population size, million									
Less than one	52,009	495,319	10.7 (10.6–10.8)	39,135	271,434	15.0 (14.8–15.1)	12874	223,885	6.31 (6.18–6.43)
One to five	88,033	799,101	11.0 (10.9–11.1)	65,454	430,926	15.0 (14.9–15.1)	22579	368,175	6.90 (6.80–7.01)
More than five	85,198	748,871	11.5 (11.4–11.5)	61,910	383,785	15.8 (15.6–15.9)	23288	365,086	7.01 (6.91–7.12)
Geographical region									
Northern	24,733	275,820	9.12 (8.99–9.24)	18,173	143,232	12.8 (12.6–13.0)	6560	132,587	5.31 (5.17–5.45)
Eastern	70,280	711,251	10.1 (10.0–10.2)	52,167	371,155	14.0 (13.8–14.1)	18,113	340,097	6.07 (5.96–6.17)
South-Central	64,429	501,046	12.6 (12.5–12.7)	47,824	273,302	17.0 (16.8–17.1)	16,605	227,744	8.03 (7.88–8.18)
Northeast	19,678	181,717	11.3 (11.1–11.4)	14,364	95,348	15.7 (15.5–16.0)	5314	86,368	6.65 (6.45–6.85)
Northwest	10,166	114,145	8.64 (8.45–8.83)	7949	66,444	11.9 (11.6–12.2)	2217	47,701	5.27 (5.02–5.52)
Southwest	35,954	259,312	14.2 (14.0–14.3)	26,022	136,663	19.0 (18.8–19.3)	9932	122,649	9.14 (8.93–9.34)
Annual average temperature, °C									
3–13	68,694	656,087	10.6 (10.5–10.7)	50,723	347,944	14.7 (14.6–14.9)	17,971	308,142	6.40 (6.30–6.51)
14–16	79,362	709,522	10.8 (10.7–10.9)	55,175	370,885	14.9 (14.8–15.0)	20,075	338,637	6.56 (6.46–6.67)
17–25	77,184	677,682	11.9 (11.8–12.0)	60,601	367,315	16.1 (16.0–16.2)	20,695	310,368	7.51 (7.39–7.63)
BMI, kg/m^2^									
<18.5	4429	91,279	5.17 (4.95–5.39)	2438	29,872	7.02 (6.66–7.37)	1991	61,407	3.25 (3.01–3.50)
18.5–23.9	75,975	947,877	8.69 (8.62–8.75)	51,057	415,393	11.9 (11.8–12.0)	24,918	532,485	5.35 (5.27–5.44)
24–27.9	83,158	587,263	13.8 (13.7–13.9)	65,609	385,124	17.8 (17.6–17.9)	17,549	202,139	9.71 (9.54–9.88)
≥28.0	33,786	179,115	18.6 (18.3–18.8)	26,594	126,051	22.1 (21.8–22.4)	7192	53,064	14.9 (14.5–15.3)
eGFR, mL/min per 1.73 m^2^									
<60	2068	11,343	17.9 (15.5–20.3)	1240	5461	25.1 (20.7–29.5)	828	5882	10.5 (8.8–12.1)
60–89	38,786	302,200	12.6 (12.4–12.8)	28,941	182,517	17.4 (17.1–17.7)	9845	119,682	7.65 (7.47–7.82)
≥90	178,214	1,678,707	10.4 (10.3–10.5)	131,754	871,821	14.3 (14.2–14.4)	46,460	806,886	6.35 (6.26–6.43)
Hypertension									
Yes	54,465	385,767	13.7 (13.5–13.9)	42479	264794	17.8 (17.6–18.1)	11,986	120,973	9.48 (9.18–9.77)
No	16,0947	1,571,874	10.3 (10.3–10.4)	116649	774305	14.3 (14.2–14.4)	44,298	797,569	6.23 (6.14–6.31)
Dyslipidemia									
Yes	117,805	908,516	12.3 (12.2–12.3)	91634	540,853	16.7 (16.6–16.9)	26,171	367,663	7.63 (7.53–7.73)
No	10,5542	1,119,155	10.0 (10.0–10.1)	73431	537,058	13.7 (13.6–13.8)	32,111	582,097	6.25 (6.17–6.33)
Fat liver disease									
Yes	80,522	615,007	16.6 (16.4–16.7)	82,659	431,674	20.3 (20.2–20.5)	21,068	183,333	12.7 (12.5–12.9)
No	115,173	1,374,093	8.96 (8.90–9.01)	79,353	626,689	12.4 (12.3–12.5)	35,820	747,403	5.43 (5.36–5.50)

Incidence rates were standardized for age and sex, incidence rates in different age group were standardized for sex only, incidence rates in different sex were standardized for age only, sex-specific incidence rates in different age group were without standardized. BMI: body mass index; eGFR: estimated glomerular filtration rate. *p* < 0.001 for all subgroup comparisons.

**Table 3:** Odds ratios (95%CIs) for associations between risk factors and hyperuricemia.

	Total		Men		Women	
	Unadjusted	Multivariable Adjusted	Unadjusted	Multivariable Adjusted	Unadjusted	Multivariable Adjusted
Age, years						
18–39	1.00	1.00	1.00	1.00	1.00	1.00
40–59	0.84 (0.83–0.85)	0.66 (0.65–0.67)	0.73 (0.72–0.74)	0.62 (0.61–0.62)	1.05 (1.03–1.07)	0.77 (0.76–0.79)
≥60	0.89 (0.87–0.90)	0.63 (0.61–0.64)	0.58 (0.57–0.59)	0.47 (0.46–0.48)	1.85 (1.81–1.90)	0.97 (0.94–1.01)
Sex						
Women	1.00	1.00	-	-	-	-
Men	2.79 (2.76–2.81)	2.20 (2.18–2.23)	-	-	-	-
Urban population size, million						
Less than one	1.00	1.00	1.00	1.00	1.00	1.00
One to five	1.07 (1.06–1.08)	1.19 (1.18–1.21)	1.08 (1.07–1.10)	1.16 (1.14–1.18)	1.09 (1.06–1.11)	1.24 (1.21–1.27)
More than five	1.12 (1.11–1.13)	1.11 (1.09–1.12)	1.17 (1.16–1.19)	1.09 (1.07–1.11)	1.14 (1.12–1.17)	1.11 (1.08–1.15)
Geographical region						
Northern	1.00	1.00	1.00	1.00	1.00	1.00
Eastern	1.11 (1.09–1.12)	1.05 (1.03–1.08)	1.12 (1.10–1.14)	1.07 (1.04–1.10)	1.08 (1.05–1.11)	1.03 (0.98–1.08)
South-Central	1.49 (1.46–1.51)	1.51 (1.48–1.55)	1.45 (1.43–1.48)	1.48 (1.43–1.52)	1.50 (1.45–1.54)	1.65 (1.58–1.73)
Northeast	1.21 (1.19–1.23)	1.21 (1.18–1.24)	1.19 (1.17–1.22)	1.20 (1.17–1.23)	1.24 (1.19–1.29)	1.26 (1.20–1.31)
Northwest	0.98 (0.96–1.00)	1.01 (0.98–1.04)	0.93 (0.90–0.95)	0.97 (0.94–1.00)	0.92 (0.88–0.97)	1.11 (1.05–1.18)
Southwest	1.63 (1.60–1.66)	1.78 (1.74–1.82)	1.62 (1.58–1.65)	1.72 (1.67–1.76)	1.68 (1.63–1.74)	1.94 (1.86–2.01)
Annual average temperature, °C						
3–13	1.00	1.00	1.00	1.00	1.00	1.00
14–16	0.97 (0.96–0.98)	0.96 (0.93–0.98)	0.97 (0.95–0.98)	0.94 (0.92–0.97)	0.94 (0.92–0.96)	0.98 (0.94–1.02)
17–25	1.21 (1.20–1.22)	1.17 (1.14–1.20)	1.23 (1.21–1.24)	1.12 (1.09–1.15)	1.22 (1.20–1.25)	1.31 (1.25–1.36)
BMI, kg/m^2^						
<18.5	1.00	1.00	1.00	1.00	1.00	1.00
18.5–23.9	1.71 (1.66–1.76)	1.66 (1.61–1.72)	1.57 (1.51–1.64)	1.68 (1.60–1.75)	1.46 (1.40–1.53)	1.51 (1.44–1.59)
24–27.9	3.23 (3.14–3.34)	2.42 (2.34–2.51)	2.30 (2.21–2.40)	2.31 (2.21–2.42)	2.83 (2.70–2.97)	2.42 (2.30–2.55)
≥28.0	4.56 (4.41–4.71)	2.95 (2.85–3.06)	2.99 (2.87–3.13)	2.74 (2.62–2.88)	4.67 (4.44–4.92)	3.29 (3.11–3.49)
eGFR, mL/min per 1.73 m^2^						
<60	1.00	1.00	1.00	1.00	1.00	1.00
60–89	0.68 (0.65–0.71)	0.54 (0.51–0.57)	0.66 (0.62–0.70)	0.52 (0.48–0.56)	0.56 (0.52–0.61)	0.61 (0.56–0.66)
≥90	0.55 (0.53–0.58)	0.45 (0.43–0.48)	0.63 (0.59–0.67)	0.44 (0.41–0.47)	0.38 (0.36–0.41)	0.47 (0.43–0.51)
Hypertension						
No	1.00	1.00	1.00	1.00	1.00	1.00
Yes	1.43 (1.42–1.45)	1.15 (1.13–1.16)	1.07 (1.06–1.08)	1.11 (1.09–1.13)	1.86 (1.82–1.90)	1.29 (1.26–1.33)
Dyslipidemia						
No	1.00	1.00	1.00	1.00	1.00	1.00
Yes	1.44 (1.43–1.45)	1.19 (1.17–1.20)	1.29 (1.28–1.31)	1.17 (1.16–1.19)	1.32 (1.30–1.34)	1.20 (1.18–1.23)
Fat liver disease						
No	1.00	1.00	1.00	1.00	1.00	1.00
Yes	2.21 (2.19–2.23)	1.55 (1.53–1.57)	1.63 (1.61–1.65)	1.43 (1.41–1.45)	2.57 (2.53–2.62)	1.86 (1.82–1.91)

BMI: body mass index; eGFR: estimated glomerular filtration rate. Multivariable adjusted model adjusted for age, sex, urban population size, geographical region, annual average temperature, BMI, eGFR, hypertension, dyslipidemia and fat liver.

## Data Availability

All data used to support the findings of this study may be released upon application to the Meinian Institute of Health (Beijing, China), which can be contacted through Prof. Yi Ning (email: Yi.Ning@MeinianResearch.com).

## Supplementary Materials

The following are available online at https://www.mdpi.com/1660-4601/18/5/2360/s1, Table S1: Incidence of hyperuricemia by age groups; Table S2: Incidence of hyperuricemia by province.
