# Supplementary material for: Incidence and Risk Factors of Hyperuricemia among 2.5 Million Chinese Adults during the Years 2017–2018
Source: Int J Environ Res Public Health. 2021 Feb 28;18(5):2360. doi: 10.3390/ijerph18052360 (PMC7957707; doi:10.3390/ijerph18052360)
Supplement: Supplementary file 1 [file ijerph-18-02360-s001.pdf]

Table S1. Incidence of hyperuricemia by age groups.

	Incidence per 100 Person-Years (95% Confidence Interval)	
Age, years	Men	Women
18–22	20.8 (20.1–21.4)	8.92 (8.45–9.39)
23–27	19.0 (18.8–19.3)	6.64 (6.50–6.78)
28–32	17.8 (17.6–17.9)	5.71 (5.60–5.82)
33–37	16.9 (16.7–17.0)	4.95 (4.85–5.06)
38–42	15.5 (15.3–15.7)	4.34 (4.23–4.45)
43–47	14.3 (14.1–14.5)	4.81 (4.69–4.93)
48–52	13.2 (13.0–13.4)	6.79(6.62–6.95)
53–57	12.3(12.1–12.6)	8.18 (7.97–8.39)
58–62	11.0 (10.8–11.3)	8.40 (8.14–8.66)
63–67	10.7 (10.4–11.0)	9.74 (9.40–10.09)
68–72	11.1 (10.7–11.6)	10.7 (10.2–11.3)
73–77	12.0 (11.4–12.5)	13.1 (12.4–13.9)
≥78	12.2 (11.5–12.8)	14.1 (13.1–15.1)

Table S2. Incidence of hyperuricemia by province.

	Incidence per 100 Person-Years (95% Confidence Interval)	
Province	Men	Women
Guangdong	22.0 (21.6–22.4)	11.7 (11.3–12.1)
Yunnan	20.4 (19.9–21.0)	11.9 (11.4–12.4)
Qinghai	25.7 (22.2–29.2)	11.7 (9.2–14.1)
Fujian	19.8 (18.7–20.9)	10.3 (9.1–11.6)
Guizhou	17.0 (16.4–17.7)	8.44 (7.85–9.04)
Sichuan	18.2 (17.9–18.5)	8.05 (7.80–8.31)
Liaoning	15.8 (15.5–16.1)	6.80 (6.58–7.02)
Hubei	15.5(15.1–15.9)	6.67 (6.34–7.00)
Hunan	16.2 (15.6–16.8)	7.30 (6.82–7.79)
Heilongjiang	17.0 (15.9–18.1)	5.28 (4.51–6.06)
Jiangxi	17.6 (16.9–18.3)	6.71 (6.16–7.26)
Jilin	14.8 (14.1–15.6)	6.36 (5.82–6.91)
Hainan	13.4 (12.7–14.1)	5.54 (4.86–6.23)
Zhejiang	12.0 (11.6–12.4)	4.82 (4.44–5.20)
Jiangsu	15.3 (15.0–15.6)	7.38 (7.04–7.72)
Shanxi	15.2 (14.7–15.7)	6.55 (6.16–6.95)
Shandong	14.5 (14.2–14.8)	5.98 (5.77–6.20)
Hebei	15.1 (14.6–15.6)	6.30 (5.96–6.65)
Henan	14.0 (13.7–14.3)	5.73 (5.51–5.95)
Anhui	12.7 (12.3–13.1)	5.35 (4.99–5.72)
Shaanxi	10.6 (10.2–11.1)	4.44 (4.08–4.79)
Gansu	11.3 (10.7–11.8)	4.01 (3.50–4.52)
Beijing	10.0 (9.6–10.4)	5.07 (4.78–5.36)
Tianjin	11.3 (10.9–11.7)	4.23 (3.98–4.48)
Shanghai	12.6 (12.4–12.9)	5.71 (5.55–5.87)
Chongqing	22.4 (21.5–23.3)	10.5 (9.5–11.5)
Guangxi	16.1 (15.4–16.7)	7.79 (7.19–8.40)
Ningxia	11.7 (10.7–12.7)	7.92 (6.72–9.11)
Xinjiang	13.6 (13.0–14.2)	6.01 (5.48–6.54)
Inner Mongolia	12.3 (11.7–12.8)	4.43 (4.03–4.84)

Incidence rates were standardized for age.
